# Construction of an *Escherichia coli* cell factory to synthesize taxadien-5α-ol, the key precursor of anti-cancer drug paclitaxel

**DOI:** 10.1186/s40643-022-00569-5

**Published:** 2022-08-13

**Authors:** Qing-Yang Wu, Zheng-Yu Huang, Jin-Yi Wang, Hui-Lei Yu, Jian-He Xu

**Affiliations:** State Key Laboratory of Bioreactor Engineering, Shanghai Collaborative Innovation Centre for Biomanufacturing, College of Biotechnology, East China University of Science and Technology, Shanghai, 200237 People’s Republic of China

**Keywords:** Oxygenated taxanes, Metabolic engineering, Taxadiene-5α-hydroxylase, Fusion protein, Mevalonate pathway, Paclitaxel, Escherichia coli

## Abstract

Paclitaxel (Taxol™), an alkaloid of diterpenoid family, is one of the most widely used anti-cancer drugs due to its effectiveness against a variety of tumors. Rather than directly extraction and chemical synthesis of paclitaxel or its intermediates from yew plants, construction of a microbial cell factory for paclitaxel biosynthesis will be more efficient and sustainable. The challenge for biosynthesis of paclitaxel lies on the insufficient precursor, such as taxadien-5α-ol. In this study, we report a recombinant *Escherichia coli* strain constructed with a heterologous mevalonate pathway, a taxadiene synthase from yew, and a cytochrome P450-mediated oxygenation system for the de novo production of taxadien-5α-ol, the first product of the multi-step taxadiene oxygenation metabolism*.* The key enzymes including taxadiene synthases and cytochrome P450 reductases were screened, and the linker for fusing taxadiene-5α-hydroxylase with its reductase partner cytochrome P450 reductase was optimized. By reducing the metabolic burden and optimizing the fermentation conditions, the final production of total oxygenated taxanes was raised up to 27 mg L^−1^ in a 50-mL flask cultivation, of which the yield of taxadien-5α-ol was 7.0 mg L^−1^, representing approximately a 12-fold and 23-fold improvements, respectively, as compared with the initial titers. The engineered MVA pathway for the overproduction of terpenoid precursors can serve as an efficient platform for the production of other valuable terpenoids.

**Graphical Abstract:**

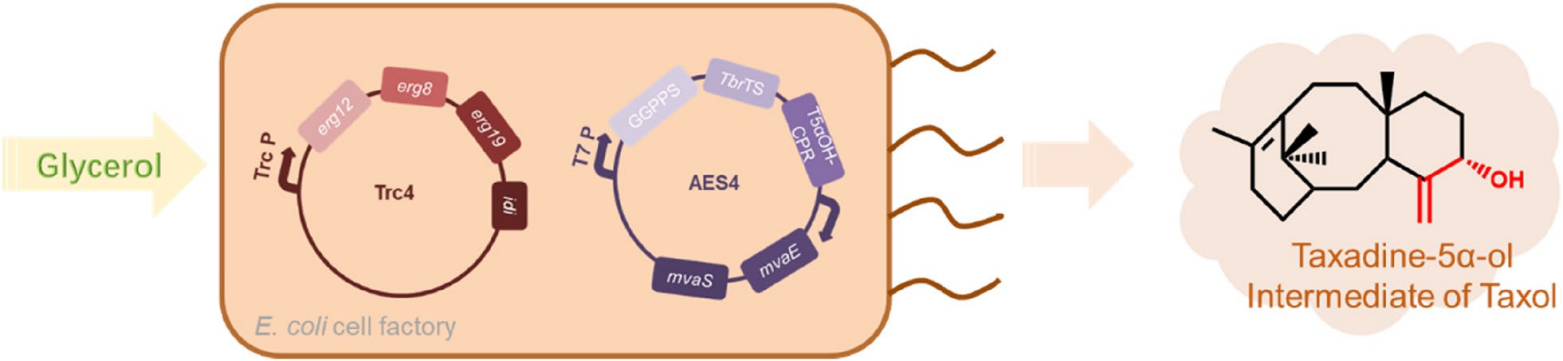

**Supplementary Information:**

The online version contains supplementary material available at 10.1186/s40643-022-00569-5.

## Highlights

- We report a recombinant *Escherichia coli* BL21(DE3) strain for de novo production of taxadien-5α-ol, the key precursor of paclitaxel.
- Through screening of key enzymes and the fermentation condition optimization, the final production of total oxygenated taxanes was raised up to 27 mg L^−1^ in 50-mL flask cultivation, of which the yield of taxadien-5α-ol was 7.0 mg L^−1^, representing approximately a 12-fold and 23-fold improvements, respectively.
- It is believed that the strategy used in this study will guide in the synthesis of terpenoids.

**Supplementary Information:**

The online version contains supplementary material available at 10.1186/s40643-022-00569-5.

## Introduction

Paclitaxel, originally isolated from the bark of *Taxus brevifolia*, is a highly effective anti-cancer drug with broad anti-cancer activity, which has been used widely in clinical treatments (Harry and Long 1994; Holmes et al. 1991). The yield of extraction from the bark of yew plants was so low that production of 1 g paclitaxel requires at least three mature yew trees (Nadeem et al. 2002; Wheeler et al. 1992), which is neither environmentally friendly nor conducive to sustainable development. Chemists have achieved great progresses in obtaining paclitaxel from simple building blocks via different synthetic routes, but with the drawbacks of extremely low yields and multiple steps (Danishefsky et al. 1996; Holton et al. 1994; Hu et al. 2021; Nicolaou et al. 1994). The semi-synthesis through extracting of the intermediates, baccatin III and 10-deacetylbaccatin III, from the renewable needles of yew plants and then transforming them into paclitaxel by chemical methods is currently the main approach for industrial production (Baloglu and Kingston 1999). Although semi-synthesis is highly productive and relatively sustainable, it is still limited by the growth of yew plants. With the rapid development of synthetic biology and metabolic engineering, heterologous expression of complex natural product biosynthetic pathways via the construction of fast-growing microbial cell factories provides a promising and sustainable solution (Hussain et al. 2021; Navale et al. 2021; Walls et al. 2021). The biosynthesis of paclitaxel and its intermediates through constructing microbial cell factories has become a popular research hotspot (Sanchez-Munoz et al. 2020; Xiong et al. 2021).

Paclitaxel is a secondary metabolite of terpenoid family that requires the universal precursors of all terpenoids, isopentenyl diphosphate (IPP) and dimethylallyl diphosphate (DMAPP), as C_5_ building blocks which are synthesized from mevalonate (MVA) pathway or 2-C-methyl-D-erythritol-4-phosphate (MEP) pathway (Withers and Keasling 2007; Yamada et al. 2015). The C_5_ terpenoid building blocks (IPP and DMAPP) were then condensed by geranylgeranyl diphosphate synthase (GGPPS) to generate a C_20_ diterpene, namely geranylgeranyl diphosphate (GGPP). The first step in the biotransformation of GGPP is its cyclization catalyzed by taxadiene synthase (TS), yielding taxadiene [taxa‐4(5),11(12)-diene] and its isomer *iso*‐taxadiene [taxa‐4(20),11(12)-diene] (Croteau et al. 2006; Hezari et al 1995; Huang et al. 2021; Soliman and Tang 2015; Wildung and Croteau 1996; Williams et al. 2000). Both the compounds can be catalyzed and modified by the subsequent taxadiene-5α-hydroxylase (CYP725A4, T5αOH) to yield a variety of mono- and di-oxygenated taxanes (totally referred as ‘oxygenated taxanes’), and only taxadien-5α-ol can be used as the precursor of paclitaxel, which suggests that the supply of taxadien-5α-ol is important for biosynthesis for paclitaxel (Jennewein et al. 2004; Rouck et al. 2017; Schoendorf et al. 2001; Xiong et al. 2021). Subsequently, at least 17 steps of enzymatic catalysis are required to obtain paclitaxel, of which some cytochrome P450s remain to be overexpressed and characterized (Cheng et al. 2021; Croteau et al. 2006; Mutanda et al. 2021; Xiong et al. 2021).

Although taxadiene can be produced with a high titer by microbial cell factories, the subsequent cytochrome P450-based oxidation chemistry still presents a significant challenge. 1 g L^−1^ taxadiene was produced in an engineered *Escherichia coli* (strain no. MG1655) using a multivariate-modular pathway engineering approach, however, the introduction of T5αOH and cytochrome P450 reductase (CPR) disrupted the carefully regulated metabolic balance, yielding only 116 mg L^−1^ oxygenated taxanes (Ajikumar et al. 2010). The expression of T5αOH and its reductase partner in *E. coli* was optimized by *N*-terminal modification, which realized a fivefold enhancement in the titer of oxygenated taxanes, reaching 570 mg L^−1^. This indicated that the prokaryotic system is also suitable for P450-based oxidation chemistry, but requires careful regulation (Biggs et al. 2016a). Since yeast system has a natural endomembrane system, it is considered to be a more suitable microbial chassis for P450 chemistry. Using *E. coli* and *Saccharomyces cerevisiae* in a consortium together combines dual properties of rapid production of taxadiene in *E. coli* with efficient oxygenation of taxadiene by *S. cerevisiae*. Through this mixed system of *E. coli* and *S. cerevisiae*, the oxygenated taxanes titer reached 33 mg L^−1^ (Zhou et al. 2015). However, the mass transfer obstacle between *E. coli* module and *S. cerevisiae* module may lead to a decrease in the titers of oxygenated taxanes. By constructing an efficient *S. cerevisiae* cell factory to de novo synthesize the oxygenated taxanes, the final titer reached 98.9 mg L^−1^ in a 1-L microbioreactor, which is the highest titer reported in yeast (Walls et al. 2021). Plants can also be an efficient chassis to produce oxygenated taxanes. With a chloroplastic compartmentalized strategy, the synthesis of taxadien-5α-ol in *Nicotiana benthamiana* was achieved for the first time, yielding 1.3 μg g^−1^ cell fresh weight (Li et al. 2019). Although eukaryotic systems are suitable for the expression of P450s chemistry due to their endometrial system, their yields are still inferior to prokaryotic host *E. coli*.

In this study, we constructed an *E. coli* cell factory capable of the de novo production of oxygenated taxanes from simple carbon sources such as glycerol. We identified a taxadiene synthase and CPR, designed a fusion protein of T5αOH-CPR, and optimized the expression of the fusion protein to construct an efficient synthetic pathway (Ajikumar et al. 2010; Wang et al. 2021). By reducing the metabolic burden of the engineered *E. coli* cells, introducing the heterologous MVA pathway and optimizing the culture conditions, final yields of the oxygenated taxanes and taxadien-5α-ol were improved, respectively, as compared to the initial titers.

## Materials and methods

### Bacterial strains, genes and vectors

*Escherichia coli* BL21 (DE3) (Novagen, Germany) was used for gene cloning and expression. The gene sequences of geranylgeranyl diphosphate synthase (GGPPS), taxadiene-5α-hydroxylase (T5αOH) and cytochrome P450 reductase (CPR) from *Taxus canadensis* (Genbank accession numbers: AF081514, AY289209 and AY571340), the taxadiene synthases *Tbr*TS, *Tba*TS and *Tw*TS from *Taxus brevifolia*, *Taxus baccata* and *Taxus wallichiana* (Genbank accession numbers: U48796, AY424738 and DQ092389), *mvaE* and *mvaS* from *Enterococcus faecalis* (Genbank accession numbers: AAG02439 and AAG02438), *erg12*, *erg8*, *erg19* and *idi* were from *Saccharomyces cerevisiae* (Genbank accession numbers: QHB10938, QHB10950, KAG2512828 and QHB12144) were synthesized and codon-optimized for *E. coli* by GenScript (Nanjing, China). The P450s reductase partner ATR from *Arabidopsis thaliana* was preserved in our laboratory. Vectors pET21a( +), pRSFDuet-1, pTrcHis2B and pACYCDuet-1 (Novagen, Germany) were used for gene expression.

### Genes cloning and plasmids construction

All plasmids were constructed by ClonExpress^®^ Ultra One Step Cloning Kit (Vazyme, China). All primers used are listed in Additional file 1: Table S2. Nucleotides corresponding to the 98 and 60 N-terminal amino acids of GGPPS and three TSs were removed. For transmembrane engineering, truncation at 24 amino acid residues on the *N*-terminal transmembrane region of T5αOH and 74 amino acid residues on the *N*-terminal transmembrane region of CPR and ATR was performed. The removal of 24 residue *N*-terminal amino acid of T5αOH, incorporation of the bovine 17α hydroxylase *N*-terminal 8 residue peptide MALLLAVF to the truncated *N*-terminal and GSTGS linker was added (Ajikumar et al. 2010). The p40T7-*dxs-idi* plasmid of MEP pathway refers to the literature (Du et al. 2014). The pRSFDuet-1-*Tbr*TS-GGPPS (Additional file 1: Table S1) was constructed by primers TG-MF/R to clone genes of TS-GGPPS and primers TG-ZF/R to clone linearized vector from pRSFDuet-1. pACYCDuet-1-T5αOH-CPR was constructed by primers T5-MF/R and CPR-MF/R to clone genes of T5αOH and CPR and primers TC-MF/R to clone linearized vector pACYCDuet-1. As for the MVA pathway, pTrcHis2B-*erg12-erg8-erg19-idi* plasmid was constructed and named as TrcE operon, and each two genes were linked by RBS sequences (5′-GTATAAGAGGAGGTAAAAAAAC-3′). The construction of pACYCDuet-1-GGPPS-*Tbr*TS-T5αOH-CPR plasmid consistent with TrcE, which was named as pACYC-ED4, but the RBS sequence (5′-TTTAATAAGGAGATATACC-3′) was different.

### In vivo production of taxadiene and oxygenated taxanes

Then, the resulting constructs were introduced into *E. coli* BL21 (DE3), and a single colony was selected for recombinant expression. 50 mL of Terrific broth medium supplemented with 15 g L^−1^ glycerol (i.e., 12 g L^−1^ peptone, 24 g L^−1^ yeast extracts, 15 g L^−1^ glycerol, 2.31 g L^−1^ KH_2_PO_4_ and 12.54 g L^−1^ K_2_HPO_4_) containing 50 μg mL^−1^ ampicillin, 34 μg mL^−1^ chloramphenicol, and 50 μg mL^−1^ kanamycin as required in a 250 mL baffled shake flask was inoculated with 1 mL of overnight Luria–Bertani culture of freshly transformed *E. coli*. The cultures were grown at 37 ℃ and 200 rpm to an optical density at 600 nm (OD_600_) of 0.6 before inducing with the addition of IPTG (0.1 mM) and δ-aminolevulinic acid (δ-ALA, 0.2 mM) (Rahul and Emily 2018). At the same time, 5 mL *n*-dodecane was also added to the culture and the temperature was dropped to 22 ℃ for the duration of 48-h cell culture.

### Gas chromatography–mass spectrometry (GC–MS) analysis

The samples were collected from the *n*-dodecane overlayer and diluted with equal-volume methyl tert-butyl ether containing 50 mg L^−1^ of *n*-octadecane internal standard. The sample was analyzed by GC–MS with full-scan mode (*m/z* 50–350) and Rtx-5MS column (0.25 mm × 30 m, 0.25 μm film thickness) on GC–MS QP2010 SE (Shimadzu, Kyoto, Japan). The oven program was as follows: 80 ℃ (1 min hold), 80–220 ℃ (15 ℃ min^−1^), 220–250 ℃ (20 ℃ min^−1^, 1 min hold). The solvent delay was set at 8 min. The same GC protocol was followed for FID. Samples were normalized using the *n*-octadecane internal standard. Taxadiene was quantified using authentic standard. The oxygenated taxanes were quantified using taxadiene.

## Results and discussion

### Heterologous production of taxadiene and oxygenated taxanes

**Fig. 1:**
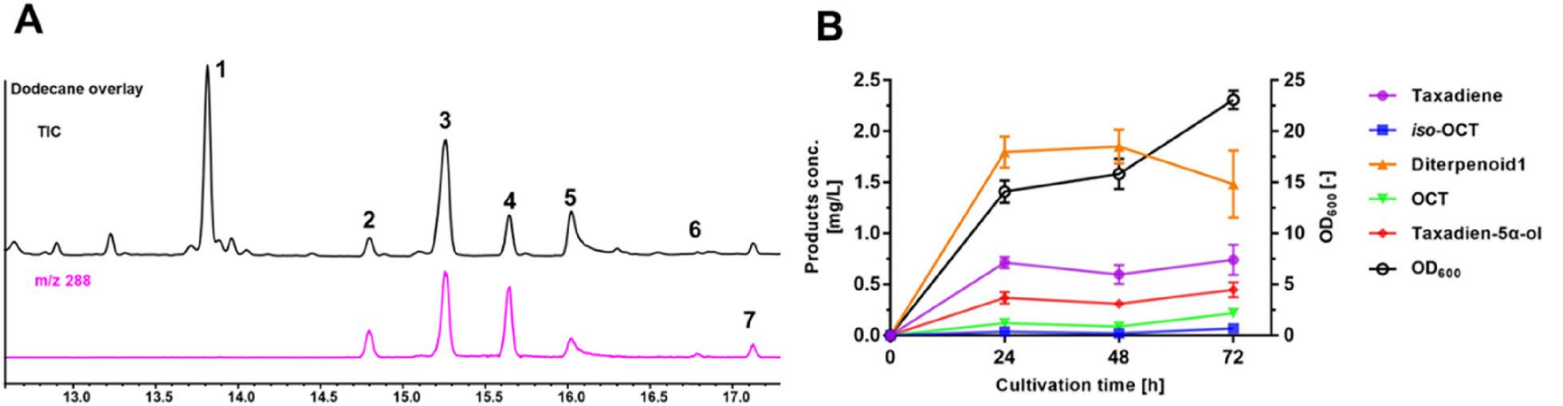
De novo synthesis of oxygenated taxanes in *E. coli* cell factories. **A** GC–MS analysis of the products of TaolE1 strain. Seven compounds are taxa‐4(5),11(12)-diene (**1**), 5(13)-oxa-3(11)-cyclotaxane (*iso*-OCT, **2**), diterpenoid1 (**3**), 5(12)-oxa-3(11)-cyclotaxane (OCT, **4**), taxadiene-5α-ol (**5**) and other unknown mono-oxygenated diterpenoids (**6, 7**). **B** Products concentrations of taxadiene and oxygenated taxanes at 48 h

We engineered a microbial cell factory by introducing a terpenoid precursor supply pathway as the upstream module and an oxygenated taxanes production pathway as the downstream module for de novo synthesis of oxygenated taxanes. The upstream terpenoid precursor supply module was constructed in p40T7-*dxs-idi* plasmid by our laboratory previously. The downstream module consisted of two plasmids, pRSFDuet-1-*Tbr*TS-GGPPS and pACYCDuet-1-T5αOH-CPR, the former generating taxadiene and the latter producing oxygenated taxanes products. The TaolE1 strain (Additional file 1: Table S1) was obtained by introducing the above three plasmids into *E. coli* BL21 (DE3). By adding 10% (v/v) *n*-dodecane, a 48-h small-scale (50-mL) fermentation experiment was carried out for the TaolE1 strain. GC–MS analysis of the *n*-dodecane phase showed that in addition to TS products, there were six mono-oxygenated taxanes products (Fig. 1A), among which 5(13)-oxa-3(11)-cyclotaxane (*iso*-OCT, **2**), 5(12)-oxa-3(11)-cyclotaxane (OCT, **4)**, and taxadien-5α-ol (**5**) were exactly consistent with the GC–MS spectra (Additional file 1: Fig. S4–S7) of the literatures (Biggs et al. 2016a, b; Li et al. 2019). We found that taxadien-5α-ol titer was higher than OCT and *iso*-OCT in this study, which was inconsistent with other literatures that OCT and *iso*-OCT as the main products were much higher than taxadien-5α-ol (Sagwan-Barkdoll and Anterola 2018). This difference in oxygenated taxanes products distribution ratio may be due to the differences in culture conditions. Edgar et al. reported that the difference in the products ratio of T5αOH may be affected by the microbial host, growth medium and extraction process (Edgar et al. 2016). After 48 h of 50-mL shake-flask fermentation, the total oxygenated taxanes titer of TaolE1 strain was 2.3 mg L^−1^, of which the titer of taxadien-5α-ol was 0.31 mg L^−1^ (Fig. 1B and Additional file 1: Table S1).

For the downstream oxygenated taxanes production module, we screened some key enzymes in hopes of improving the yields of oxygenated taxanes and taxadien-5α-ol. Two other taxadiene synthases, *Tba*TS and *Tw*TS, from *Taxus baccata* and *Taxus wallichiana*, respectively, were selected for analysis of the specific oxygenated taxanes yield. Unfortunately, these two taxadiene synthases were inferior to *Tbr*TS (Additional file 1: Fig. S1 and Table S1). Since cytochrome P450 reductase ATR derived from *Arabidopsis thaliana* (Pompon et al. 1996) was commonly used to cooperate with universal plant P450s, we also compared it with CPR derived from *Taxus cuspidate* and the results showed that CPR homologous to P450 was more effective (Additional file 1: Fig. S2 and Table S1). At present, the modification of plant P450s is generally focused on the *N*-terminal modification to improve their functional expression, but some focus on the linker part of the fusion protein of P450-CPR was optimized to improve the functional expression and its catalytic effect. Therefore, we selected five flexible linkers of (GSG)*n* (*n* = 1–5) to replace and optimize the current GSTGS linker (Wang et al. 2021). We found that these five flexible linkers can improve the soluble expression of five fusions proteins of T5αOH-CPR, but the specific oxygenated taxanes titer decreased unfortunately (Additional file 1: Fig. S3 and Table S1). This may be due to the fact that CPR and P450 could not form the correct conformation, which affected the efficient electron transfer from CPR to P450 (Wang et al. 2021).

### Reducing cell metabolic burden for higher production of oxygenated taxanes

**Fig. 2:**
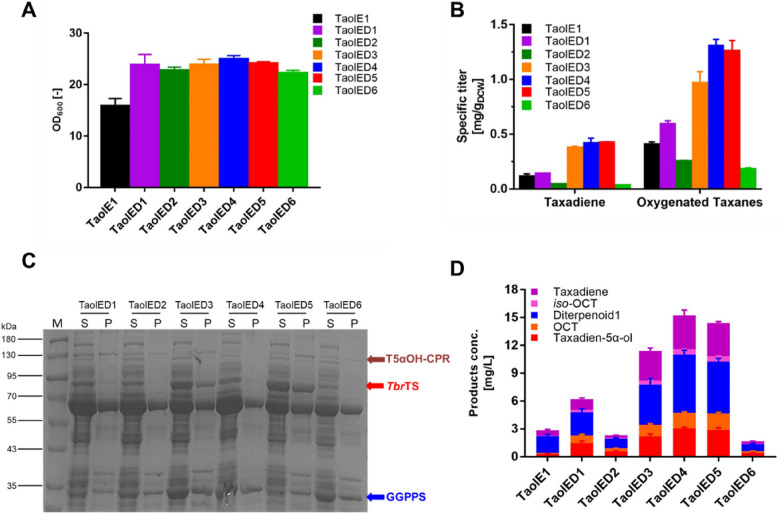
Effects of bacterial metabolic burden on the yield of oxygenated taxanes. **A** Comparison of the OD_600_ values of TaolED1-6 strains at 48 h; **B** comparison of the specific titers of TaolED1-6 strains; **C** SDS-PAGE analysis of the protein expression levels of TaolED1-6 strains; **D** comparison of the product concentrations of TaolED1-6 strains. M: marker; S: supernatant; P: precipitate

We found that despite culturing the TaolE1 strain for 48 h, the growth of the bacteria was still very poor with the OD_600_ of only 16, which affected the final oxygenated taxanes yield. The reason for this may be that the metabolic burden placed on the bacteria as a result of carrying three plasmids (Ajikumar et al. 2010). Therefore, the genes encoding GGPPS, *Tbr*TS and T5αOH-CPR located across two plasmids in the downstream oxygenated taxanes production module were reassembled into a single pACYCDuet-1. Since the different assembly sequence of genetic elements on one operon has a significant impact on the functional expression of enzymes and the resulting yield **(**Hou et al. 2021), we engineered six operon combinations (Additional file 1: Table S1) based on the different collocations of the three genes on the plasmid. Six new strains were obtained by co-expressing the upstream module p40T7-*dxs-idi*, designated TaolED*n*, *n* = 1–6 (Additional file 1: Table S1). The fermentation results showed that the OD_600_ of the six new strains was significantly higher (~ 25) than that of TaolE1 strain harboring three coexisting plasmids when cultured for 48 h (Fig. 2A). The specific oxygenated taxanes titer was significantly higher for the TaolED3-5 strains than for the TaolE1 and TaolED1-2 strains (Fig. 2B). This indicated that the heavy metabolic burden caused by too many plasmids had a significant negative impact on the products yield. However, the specific oxygenated taxanes titers of TaolED1 and TaolED6 strains were found to have decreased. Then, we detected the protein expression levels of the six strains. It showed that the soluble expression of T5αOH-CPR was very poor, which indicated that *E. coli* may not be suitable for expressing the large molecular weight of the fusion protein (127 kDa) (Fig. 2C). The soluble expression of *Tbr*TS in TaolED1-2 and TaolED6 strains was poorer compared with the other three strains, which explained the lower oxygenated taxanes yield of these three strains. The supply of taxadiene was the limiting step due to the low protein expression level of *Tbr*TS, and more taxadiene was also accumulated in the TaolED3-5 strain without further hydroxylation. The soluble expression of GGPPS in TaolED5 strain was poor compared with TaolED3-4 strains, but the production of oxygenated taxanes was not been affected. Therefore, the soluble expression level of GGPPS was not the limiting factor in the downstream module. In conclusion, the reason for the difference in oxygenated taxanes yield of TaolED1-6 strains was caused by the different expression level of *Tbr*TS, which led to the different supply capacity of taxadiene. The oxygenated taxanes titer of the optimal strain TaolED4 was 12 mg L^−1^, and the titer of taxadien-5α-ol was 3.1 mg L^−1^ (Fig. 2D).

### Introducing MVA pathway for sufficient supply of terpenoid precursors

**Fig. 3:**
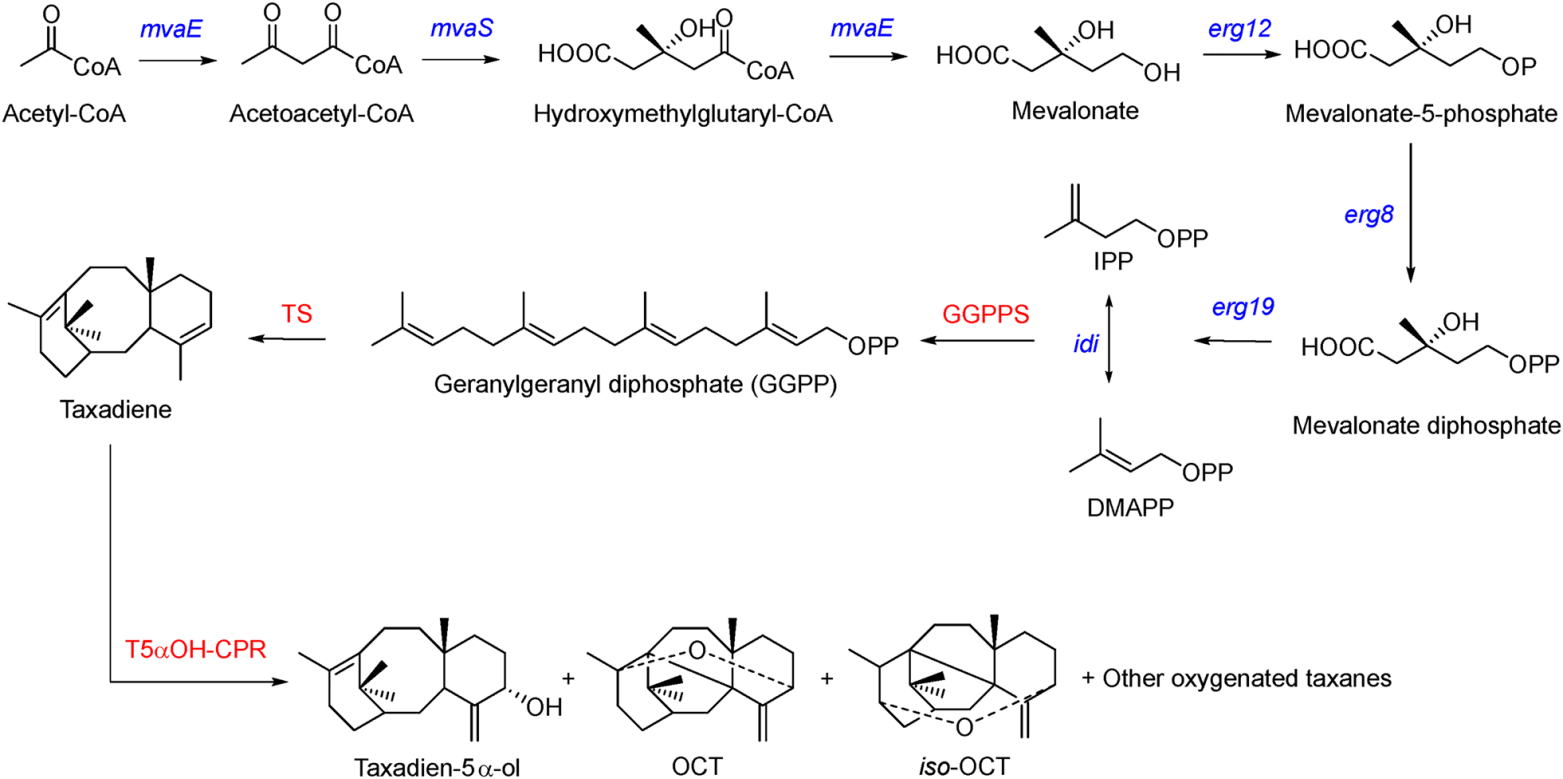
Schematic diagram of the biosynthesis of oxygenated taxanes with a heterologous MVA pathway. *mvaE*: acetoacetyl-CoA thiolase/HMG-CoA reductase gene; *mvaS*: HMG-CoA synthase gene; *erg12*: mevalonate kinase; *erg8*: phosphomevalonate kinase; e*rg19*: mevalonate pyrophosphate decarboxylase; *idi*: IPP:DMAPP isomerase; GGPPS: geranylgeranyl pyrophosphate synthase; TS: taxadiene synthase; T5αOH-CPR: the fusion protein of taxadiene-5α-hydroxylase and *Taxus*-derived cytochrome P450 reductase; OCT: 5(12)-oxa-3(11)-cyclotaxane; *iso*-OCT: 5(13)-oxa-3(11)-cyclotaxane

We already know that the oxygenated taxane products were affected by the supply of taxadiene. The insufficient supply of taxadiene may result from the lack of the terpenoid precursors IPP and DMAPP. In order to increase the supply of IPP and DMAPP, we constructed a heterologous MVA pathway that also supplied these terpenoid precursors. We introduced the genes *mvaE* and *mvaS* from *Enterococcus faecalis* and *erg12*, *erg8*, *erg19* and *idi* from *S. cerevisiae* (Fig. 3) into the engineered *E. coli* to construct a MVA pathway. We constructed three recombinant plasmids TrcE, AES4 and AES5, which the latter two plasmids have different gene assembly sequences and combined with each other to obtain two engineered strains, TaolV1 and TaolV2 (Additional file 1: Table S1).

**Fig. 4:**
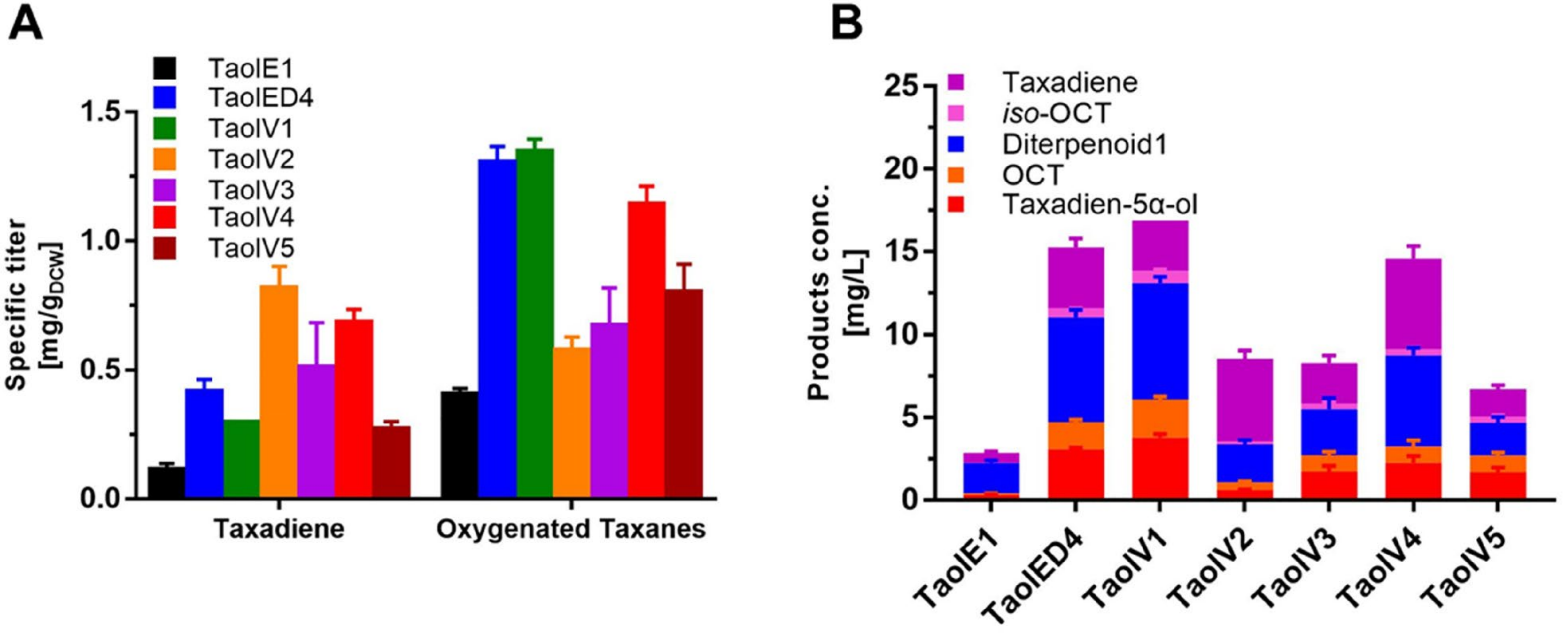
Effects of different terpenoid precursor supply pathways on the yield of oxygenated taxanes. **A** Comparison of the specific titers of TaolV1-5 strains; **B** comparison of the product concentrations of TaolV1-5 strains with those of TaolE1 and TaolED4

The TaolV1 strain adopted the genetic combination of GGPPS-*Tbr*TS-T5αOH-CPR in the TaolED4 strain, and its oxygenated taxanes titer (14 mg L^−1^) was slightly higher than that of TaolED4 strain (12 mg L^−1^) supplied by the MEP pathway **(**Fig. 4A). The supply capacity of taxadiene (the sum of the remaining taxadiene and oxygenated taxanes products) using the MVA pathway was slightly higher than that obtained by overexpressing rate-limiting enzymes in the MEP pathway. The yield of taxadien-5α-ol in TaolV1 was 3.8 mg L^−1^ (Fig. 4B), and the specific titer of TaolV2 strain was not improved compared with TaolV1 strain, which may be due to the fact that the TS gene needs to be assembled in a more advanced position on the plasmid. Since the effect of MVA pathway was slightly improved, we selected the TaolV1 strain harboring the MVA pathway for terpenoid precursors for further optimization.

We expected to enhance the existing supply of terpenoid precursors to the MVA pathway by upregulating the expression level of enzymes. By replacing the pTrcHis2B vector with the pRSFDuet-1 vector, we obtained recombinant plasmid T7E1, and the new engineered strain was designated TaolV3 (Additional file 1: Table S1). The expression strength was defined as the copy number multiplied by the promoter strength (Brosius et al. 1985; Brunner and Bujard 1987). The promoter strengths of Trc and T7 promoters were defined as 1 and 5, respectively, and the copy numbers for the TrcE and T7E1 plasmids were both 20. Therefore, the expression strengths of TrcE and T7E1 plasmids were 20 and 100, respectively (Ajikumar et al. 2010; Lv et al. 2014) (Table 1). However, the titer and specific yield of oxygenated taxanes of TaolV3 strain were not as high as TaolV1 strain (Fig. 4), indicating that the MVA pathway does not require the supply of precursors with higher expression strength. Stronger transcription may result in a heavier bacterial metabolic burden, disrupting the balance of existing terpenoid supply and downstream oxygenated taxanes production.

**Table 1:** Comparison of the expression strength of the MVA pathway

Plasmid	Vector	Promoter	Replication	Copies	Expression strength
TrcE	pTrcHis2B	Trc	pBR322	20	20
T7E1	pRSFDuet-1	T7	RSF	20	100

Considering that the yields of oxygenated taxanes produced by the MEP and MVA pathways as precursor supply pathways were highly similar, the two pathways were considered to work together to provide terpenoid precursors. The *dxs* gene was introduced into the recombinant T7E1 and TrcE1 plasmids to construct the engineered strains TaolV4 and TaolV5, respectively (Additional file 1: Table S1). The yields of taxadiene and oxygenated taxanes of TaolV5 were lower than for the TaolV4 strain. The residual taxadiene in the TaolV4 strain accounted for approximately 37% of the total supply of taxadiene, which was approximately 51% higher than that of the TaolV1 strain (Fig. 4B). This indicated that the two pathways work together to provide sufficient terpenoid precursors for the synthesis of taxadiene, but the subsequent T5αOH-CPR is not converted into oxygenated taxanes in time. The TaolV4 strain produced lower levels of oxygenated taxanes than TaolV1 strain, which may be due to the introduction of a new enzyme, *dxs*, increased the metabolic burden, thereby disrupting the metabolic balance.

### Optimizing of the fermentation conditions for TaolV1 strain

**Fig. 5:**
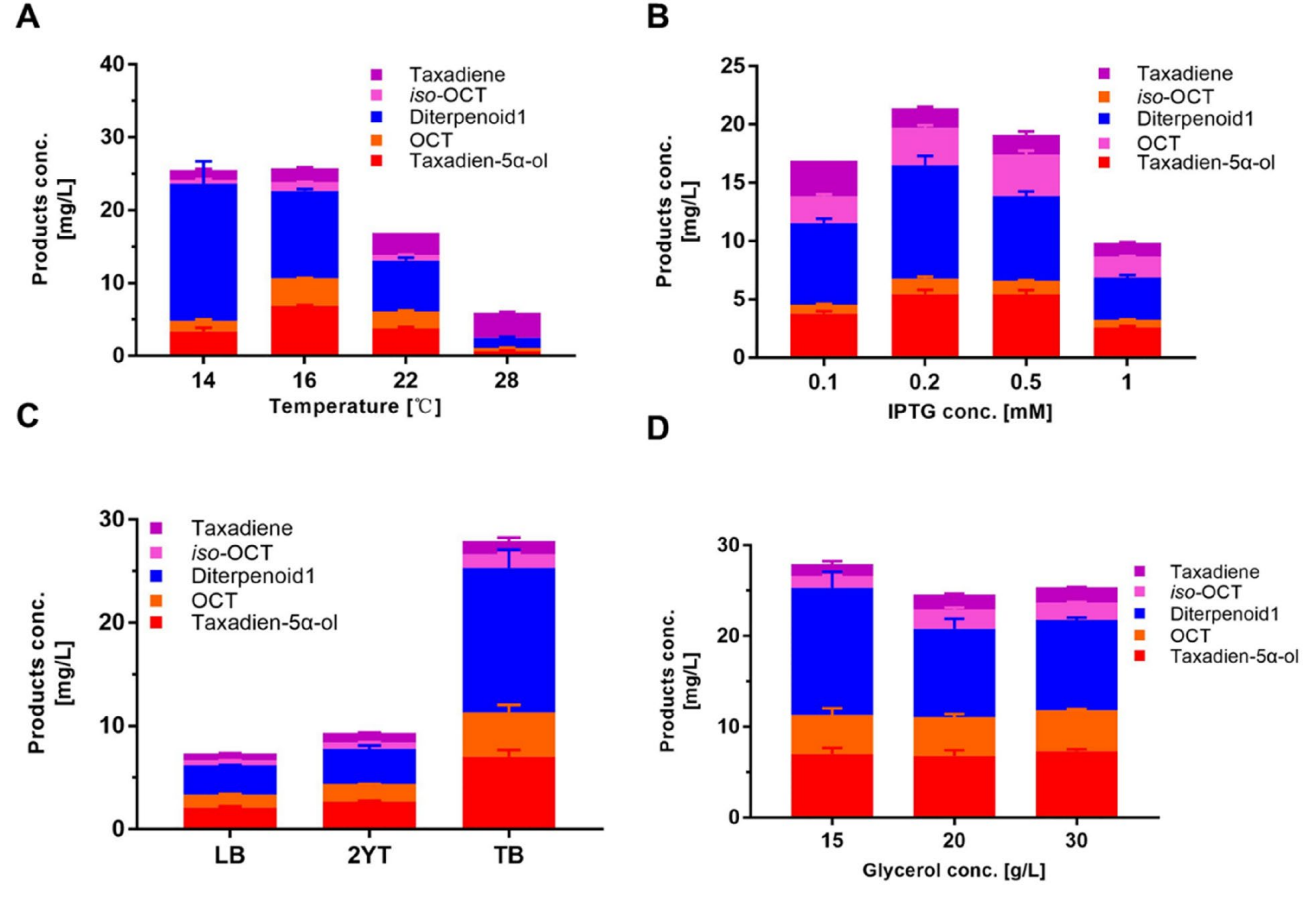
Optimization of the fermentation conditions for TaolV1 strain. **A** Temperature; **B** IPTG concentration; **C** culture medium; **D** glycerol concentration

In addition to the genetic modulation described above, we optimized the fermentation conditions to further increase the oxygenated taxanes titer of TaolV1 strain. We optimized the temperature and the IPTG concentration, respectively, and compared the effects of different media and different concentrations of carbon sources on the production of oxygenated taxanes. The yield of oxygenated taxanes at 14 °C and 16 °C was found to be essentially the same, but the yield of taxadien-5α-ol at 14 °C was extremely low (Fig. 5A), so 16 °C was selected as the optimum temperature for cultivation. This also suggested that temperatures affected the composition of various oxygenated taxanes products. Next, we analyzed the optimum IPTG concentration because high concentrations of IPTG can cause cytotoxic effects and low concentrations are not conducive to the inducible expression of target proteins. The results showed that 0.2 mM IPTG provided the highest yield of oxygenated taxanes (Fig. 5B). By comparing different growth media, nutrient-rich TB medium was found to produce higher yields of oxygenated taxanes than the LB and 2YT media with the same concentration of glycerol added (15 g L^−1^) (Fig. 5C). The oxygenated taxanes titer was not further improved by increasing the glycerol concentration (Fig. 5D). Therefore, TB medium supplemented with 15 g L^−1^ glycerol was finally selected, and 0.2 mM IPTG was added to induce proteins expression, and culturing was performed at 16 °C for 48 h. Under the above conditions, the yield of oxygenated taxanes reached 27 mg L^−1^, of which the yield of taxadien-5α-ol was 7.0 mg L^−1^, representing approximately a 12-fold and 23-fold improvement compared to the titer of the initial TaolE1 strain, respectively (Fig. 5D).

## Discussion

Construction of microbial cell factories to produce intermediates of the anti-cancer drug paclitaxel is a promising alternative method for chemical extraction from yew plants. In this study, we constructed an *E. coli* cell factory for the de novo synthesis of taxadien-5α-ol, an intermediate of paclitaxel, through the introduction of a heterologous MVA pathway, GGPPS, taxadiene synthase and T5αOH-CPR fusion protein. By optimizing the bacterial metabolic burden, the assembly sequence of genetic elements and the fermentation conditions, TaolV1 strain accumulated 27 mg L^−1^ of oxygenated taxanes in the shake-flask fermentation, of which the yield of taxadien-5α-ol was 7.0 mg L^−1^, 12-fold and 23-fold improvement compared with our initial strain, respectively. This work has thereby established an efficient microbial cell factory for the production of paclitaxel intermediates, which can also be used for extension of the paclitaxel biosynthesis pathway, such as the introduction of the next step taxadien-5α-ol *O*-acetyltransferase (TAT) (Walls et al. 2021, 2022). It also provides an *E. coli* cell factory for the production of other valuable natural terpenoid products.

Research on taxadien-5α-ol biosynthesis has achieved great success. Ajikumar et al. reported the production of 116 mg L^−1^ of oxygenated taxanes in a bioreactor through the multivariate-modular pathway engineering strategy by constructing an *E. coli* cell factory (Ajikumar et al. 2010). Biggs et al. integrated all genes on the chromosome and reported an oxygenated taxanes yield in the bioreactor scale fermentation of 570 mg L^−1^ (Biggs et al. 2016a). It showed that *E. coli* can functionally express plant P450s as an efficient cell factory for de novo production of natural terpenoids and its intermediates. Unlike previous studies that reported that OCT and *iso*-OCT were the major products of T5αOH in *E. coli* (Biggs et al. 2016a; Sagwan-Barkdoll and Anterola 2018), our results suggested that an unknown diterpenoid1 (**3**, possibly an isomer of taxadien-5α-ol) is the major product, which is consistent with the results of Walls et al. in *S. cerevisiae* (Walls et al. 2021). In addition, the desired taxadien-5α-ol in the *E. coli* host was generally a minor product in the studies mentioned above, being generated at a lower level than OCT and *iso*-OCT (Sagwan-Barkdoll and Anterola 2018). By contrast, in our study, taxadien-5α-ol was generated at a higher level than *iso*-OCT and OCT, which may be explained by the different fermentation conditions. Edgar et al. reported that taxadien-5α-ol accounted for 0–25% of all T5αOH products, depending on the microbial host, growth medium, and extraction methods.

In this study, we also compared the overexpression of rate-limiting enzymes of the MEP pathway and the introduction of the heterologous MVA pathway as terpenoid precursors supply pathways, respectively. The results showed no significant difference between these two pathways. Moreover, we found that the introduction of the MVA pathway and overexpression of the rate-limiting enzymes *dxs* and *idi* of the MEP pathway did not perform better than the MEP or MVA pathway alone. This may reflect the imbalance in bacterial metabolism caused by the introduction of more enzymes. Carrying multiple plasmids increases the metabolic burden on bacteria, which was also reflected by the findings of this study. Since the multi-plasmid expression system may overload the bacterial metabolism, it may be worth considering integrating the MVA pathway constructed in this study into the chromosome of *E. coli* MG1655 or *E. coli* DH5α which is suitable for chromosomal integration to construct an engineered *E. coli* cell factory. This strategy not only could reduce the number of plasmids carried by the bacteria and increase the yield, but also could quickly replace the downstream module for the synthesis of other natural terpenoids (Ajikumar et al. 2010; Biggs et al. 2016a).

The expression strength of the vector also affected the biosynthesis of oxygenated taxanes, and a vector with a weaker expression strength should be appropriately selected. Through our study, the yield of oxygenated taxanes were increased when the expression of taxadiene synthase was improved, which indicates that the supply of taxadiene affects the biosynthesis of oxygenated taxanes. However, the large amount of residual taxadiene suggested that the efficiency of T5αOH may be a bottleneck. To enable efficient expression of plant-derived P450s, researchers have explored various strategies, including codon optimization, *N*-terminal modification such as signal peptide introduction or transmembrane truncation, and the construction of fusion proteins (Ajikumar et al. 2010; Biggs et al. 2016a; Wang et al. 2021). When we tried linker optimization of the fusion protein T5αOH-CPR, although the (GSG)*n* (*n* = 1–5) linker improved the expression of the fusion protein, it greatly reduced the production of oxygenated taxanes. This may reflect that the two enzymes form an unfavorable conformation, leading to misorientation of the active sites in T5αOH-CPR, which ultimately affects the electron transfer efficiency of CPR to P450s (Wang et al. 2021). Therefore, in-depth structural analysis and modeling of individual enzymes are necessary for the construction of fusion proteins, in which the length and species of the linker, as well as the orientation of the enzymes are critical. After the optimization of the cultural condition, the residual taxadiene almost disappeared, which suggests that the bottleneck of the biosynthesis of oxygenated taxanes transferred to the insufficient supply of taxadiene. Hence, we still need to solve this problem by improving the catalytic efficiency of taxadiene synthase.

## Conclusions

Functional modification of terpene skeletons by cytochrome P450 hydroxylases provides the opportunity to produce a variety of biological products that are more valuable than the original terpene molecules themselves (Alonso-Gutierrez et al. 2013; Chang et al. 2007). In this study, we constructed an *E. coli* cell factory for the efficient production of diterpenoids from simple glycerol using a heterologous MVA pathway. The yields of oxygenated taxanes and taxadien-5α-ol in 50-mL shake flasks reached 27 mg L^−1^ and 7.0 mg L^−1^, respectively, with taxadien-5α-ol accounting for approximately 26% of all oxygenated taxanes products. Our engineered MVA pathway for the overproduction of terpenoid precursors can serve as an efficient platform for the production of other valuable terpenoids. The advances in metabolic engineering, as reported here, make the microbial production of terpenoid more feasible.

### Supplementary Information

**Additional file 1:** **Figure S1.** Comparison of taxadiene synthases from different sources. (A) SDS-PAGE gel of different TSs in *E. coli.* S: Supernatant; P: Precipitate. (B) Comparison of the specific oxygenated taxanes titer of TaolE1-3 strains. **Figure S2. **Comparison of cytochrome P450 reductase from different sources. (A) SDS-PAGE gel of T5αOH-ATR/CPR in *E. coli*. M: Protein marker; S: Supernatant; P: Precipitate. N.C.: Negative control. (B) The comparison of specific titer of TaolE1 and TaolE4 strains. **Figure S3. **Comparison of different linkers of T5αOH-CPR fusions. (A) SDS-PAGE gel of (GSG)n, n=1-5 linker in *E. coli*. M: Protein marker; N.C.: Negative control. S: Supernatant; P: Precipitate. (B) The comparison of specific titer of TaolE1 and TaolEGSGn strains. **Figure S4.** MS spectrum of product *iso*-OCT. Retention time of GC is 14.80 min. **Figure S5.** MS spectrum of product diterpenoid1. Retention time of GC is 15.20 min. **Figure S6. **MS spectrum of product OCT. Retention time of GC is 15.65 min. **Figure S7. **MS spectrum of product taxadien-5α-ol. Retention time of GC is 16.02 min. **Table S1.** The engineered strains constructed in this study and products titers (48 h). **Table S2.** The primers used in this study.

## Data Availability

All data generated or analyzed during this study are included in this article and its Additional file.

## Abbreviations

IPP: Isopentenyl diphosphate
DMAPP: Dimethylallyl diphosphate
GGPP: Geranylgeranyl pyrophosphate
GGPPS: Geranylgeranyl diphosphate synthase
MEP Pathway: 2-C-Methyl-D-erythritol-4-phosphate Pathway
MVA Pathway: Mevalonate pathway
Idi: Isopentenyl diphosphate isomerase
Dxs: 1-Deoxyxylulose-5-phosphate synthase
ERG12: Mevalonate kinase
ERG8: Phosphomevalonate kinase
ERG19: Mevalonate pyrophosphate decarboxylase
TS: Taxadiene synthase
T5αOH/ T5αH: Taxadiene-5α-hydroxylase
P450: Cytochrome P450 hydroxylase
CPR: Cytochrome P450 reductase
OCT: 5(12)-Oxa-3(11)-cyclotaxane
*iso-*OCT: 5(13)-Oxa-3(11)-cyclotaxane
