# Supplementary material for: Construction of an Escherichia coli cell factory to synthesize taxadien-5α-ol, the key precursor of anti-cancer drug paclitaxel
Source: Bioresour Bioprocess. 2022 Aug 13;9(1):82. doi: 10.1186/s40643-022-00569-5 (PMC10992617; doi:10.1186/s40643-022-00569-5)
Supplement: Supplementary file 1 — Additional file 1: Figure S1. Comparison of taxadiene synthases from different sources. (A) SDS-PAGE gel of different TSs in E. coli. S: Supernatant; P: Precipitate. (B) Comparison of the specific oxygenated taxanes titer of TaolE1-3 strains. Figure S2. Comparison of cytochrome P450 reductase from different sources. (A) SDS-PAGE gel of T5αOH-ATR/CPR in E. coli. M: Protein marker; S: Supernatant; P: Precipitate. N.C.: Negative control. (B) The comparison of specific titer of TaolE1 and TaolE4 strains. Figure S3. Comparison of different linkers of T5αOH-CPR fusions. (A) SDS-PAGE gel of (GSG)n, n=1-5 linker in E. coli. M: Protein marker; N.C.: Negative control. S: Supernatant; P: Precipitate. (B) The comparison of specific titer of TaolE1 and TaolEGSGn strains. Figure S4. MS spectrum of product iso-OCT. Retention time of GC is 14.80 min. Figure S5. MS spectrum of product diterpenoid1. Retention time of GC is 15.20 min. Figure S6. MS spectrum of product OCT. Retention time of GC is 15.65 min. Figure S7. MS spectrum of product taxadien-5α-ol. Retention time of GC is 16.02 min. Table S1. The engineered strains constructed in this study and products titers (48 h). Table S2. The primers used in this study. [file 40643_2022_569_MOESM1_ESM.docx]

**Supporting Information**

**Construction of an *Escherichia coli* cell factory to synthesize taxadien-5α-ol, the key precursor of anti-cancer drug paclitaxel**

**Qing-Yang Wu, Zheng-Yu Huang, Jin-Yi Wang and Hui-Lei Yu^*^ and Jian-He Xu**

^a^ State Key Laboratory of Bioreactor Engineering, Shanghai Collaborative Innovation Centre for Biomanufacturing, College of Biotechnology, East China University of Science and Technology, Shanghai 200237, P. R. China.

^*^ Corresponding author:

Prof. Hui-Lei Yu, email: [huileiyu@ecust.edu.cn](mailto:huileiyu@ecust.edu.cn)

**
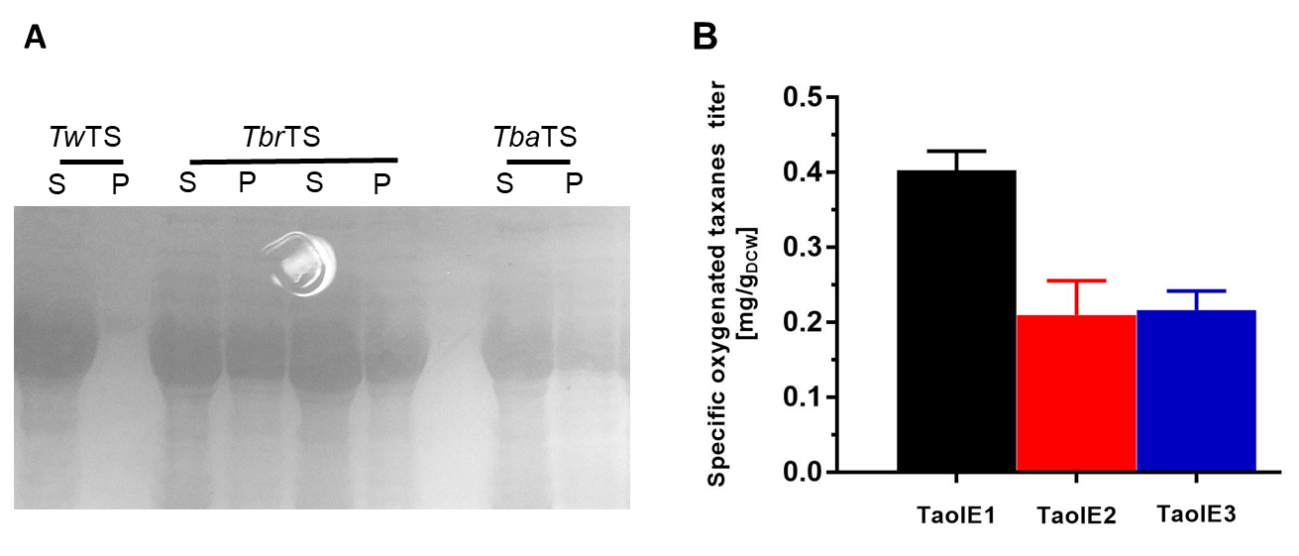
**

**Fig. S1** Comparison of taxadiene synthases from different sources. (A) SDS-PAGE gel of different TSs in *E. coli.* S: Supernatant; P: Precipitate. (B) Comparison of the specific oxygenated taxanes titer of TaolE1-3 strains.


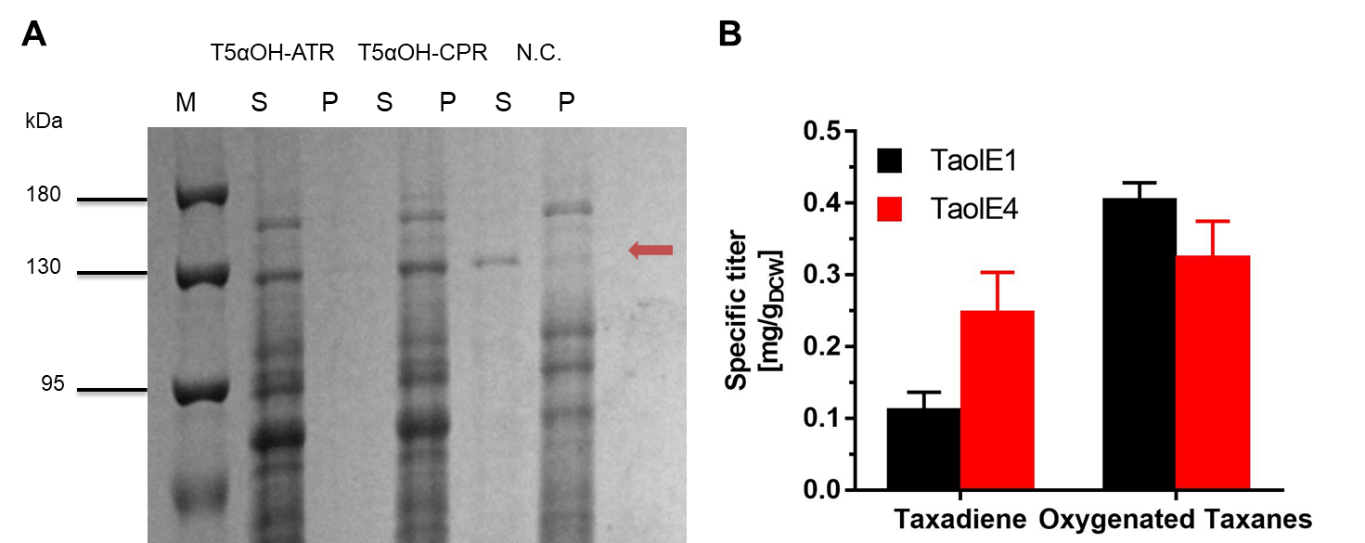


**Fig. S2** Comparison of cytochrome P450 reductase from different sources. (A) SDS-PAGE gel of T5αOH-ATR/CPR in *E. coli*. M: Protein marker; S: Supernatant; P: Precipitate. N.C.: Negative control. (B) The comparison of specific titer of TaolE1 and TaolE4 strains.


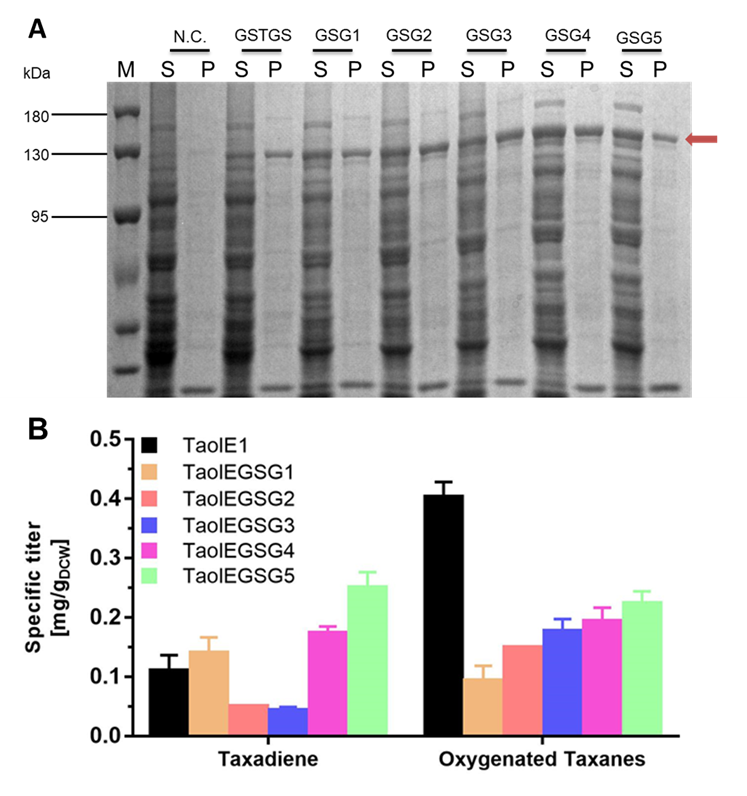


**Fig. S3** Comparison of different linkers of T5αOH-CPR fusions. (A) SDS-PAGE gel of (GSG)n, n=1-5 linker in *E. coli*. M: Protein marker; N.C.: Negative control. S: Supernatant; P: Precipitate. (B) The comparison of specific titer of TaolE1 and TaolEGSGn strains.

**Fig. S4** MS spectrum of product *iso*-OCT. Retation time of GC is 14.80 min.


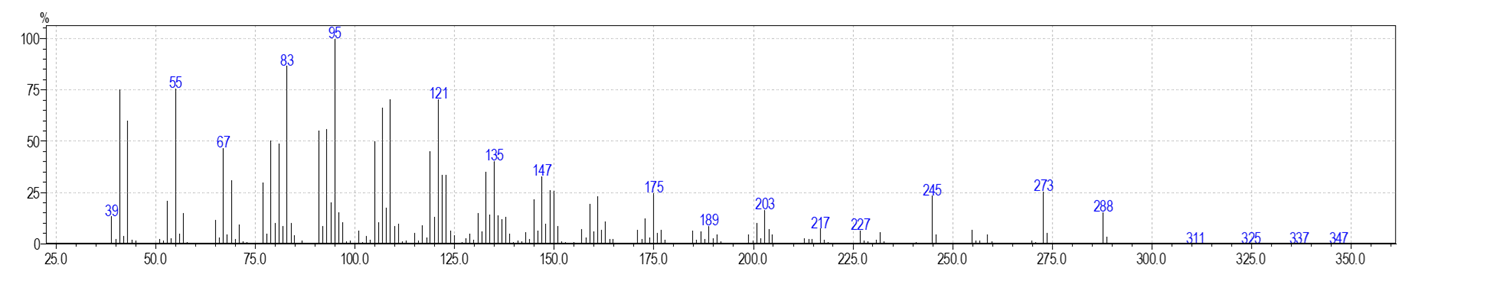


**Fig. S5** MS spectrum of product diterpenoid1. Retation time of GC is 15.20 min.


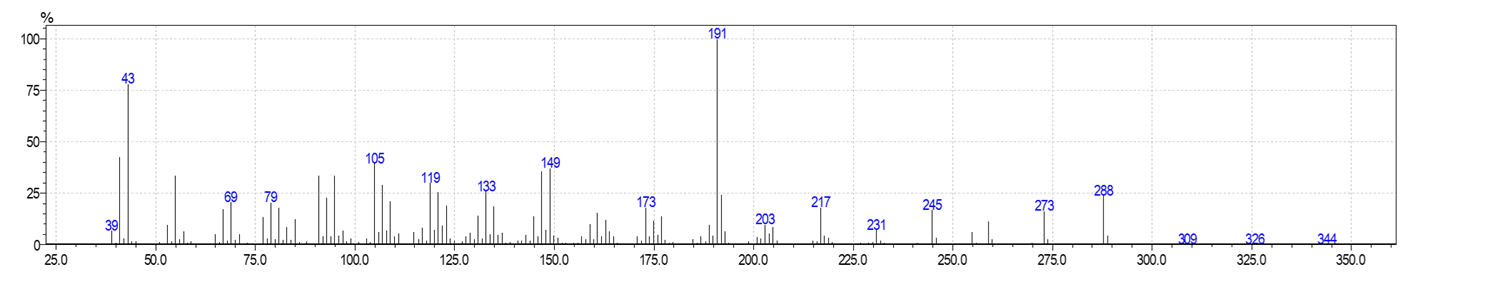


**Fig. S6** MS spectrum of product OCT. Retation time of GC is 15.65 min.


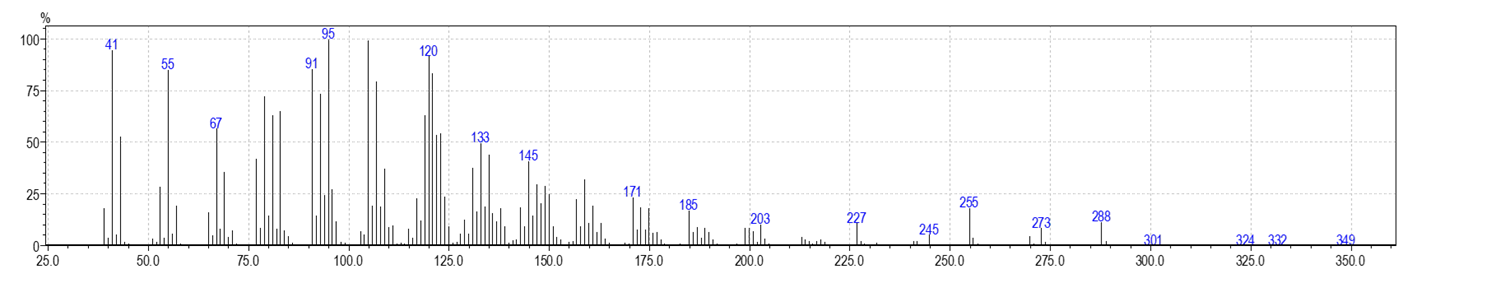


**Fig. S7** MS spectrum of product taxadien-5α-ol. Retation time of GC is 16.02 min.

**Table S1** The engineered strains constructed in this study and products titers (48 h).

| **Strains** | **Pathway engineering** | **Plasmid combinations** | **Total oxygenated taxanes titer**  **(mg L^-1^)** | **Taxadien-5α-ol titer (mg L^-1^)** | **Specific oxygenated taxanes titer**  **(mg g_dcw_^-1^)** |
| --- | --- | --- | --- | --- | --- |
| TaolE1 | 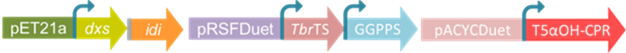 | p40T7-*dxs-idi*,  pRSFDuet-1-*Tbr*TS-GGPPS, pACYCDuet-1-T5αOH-CPR | 2.3 **^a^** | 0.31 | 0.40 |
| TaolE2 | 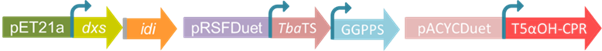 | p40T7-*dxs-idi*,  pRSFDuet-1-*Tba*TS-GGPPS, pACYCDuet-1-T5αOH-CPR | 0.95 | 0.18 | 0.21 |
| TaolE3 | 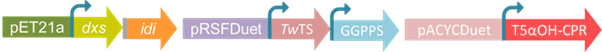 | p40T7-*dxs-idi*,  pRSFDuet-1-*Tw*TS-GGPPS, pACYCDuet-1-T5αOH-CPR | 2.1 | 0.40 | 0.22 |
| TaolE4 | 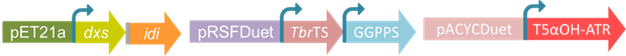 | p40T7-*dxs-idi*,  pRSFDuet-1-*Tbr*TS-GGPPS, pACYCDuet-1-T5αOH-ATR | 1.8 | 0.10 | 0.32 |
| TaolEGSG1 | **/** | p40T7-*dxs-idi*,  pRSFDuet-1-*Tbr*TS-GGPPS, pACYCDuet-1-T5αOH-(GSG)1-CPR | 0.77 | 0.27 | 0.090 |
| TaolEGSG2 | **/** | p40T7-*dxs-idi*,  pRSFDuet-1-*Tbr*TS-GGPPS, pACYCDuet-1-T5αOH-(GSG)2-CPR | 0.77 | 0.25 | 0.15 |
| TaolEGSG3 | **/** | p40T7-*dxs-idi*,  pRSFDuet-1-*Tbr*TS-GGPPS, pACYCDuet-1-T5αOH-(GSG)3-CPR | 0.96 | 0.31 | 0.18 |
| TaolEGSG4 | **/** | p40T7-*dxs-idi*,  pRSFDuet-1-*Tbr*TS-GGPPS, pACYCDuet-1-T5αOH-(GSG)4-CPR | 1.0 | 0.30 | 0.19 |
| TaolEGSG5 | **/** | p40T7-*dxs-idi*,  pRSFDuet-1-*Tbr*TS-GGPPS, pACYCDuet-1-T5αOH-(GSG)5-CPR | 1.4 | 0.39 | 0.22 |
| TaolED1 | 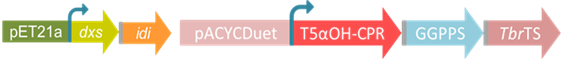 | p40T7-*dxs-idi*,  pACYC-ED1 | 5.0 | 1.5 | 0.59 |
| TaolED2 | 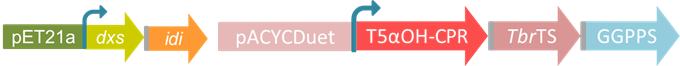 | p40T7-*dxs-idi*,  pACYC-ED2 | 2.0 | 0.59 | 0.25 |
| TaolED3 | 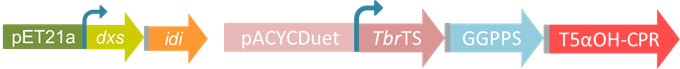 | p40T7-*dxs-idi*,  pACYC-ED3 | 8.2 | 2.2 | 0.96 |
| TaolED4 | 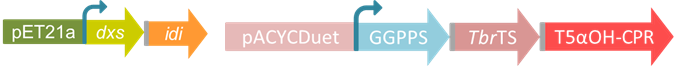 | p40T7-*dxs-idi*,  pACYC-ED4 | 12 | 3.1 | 1.3 |
| TaolED5 | 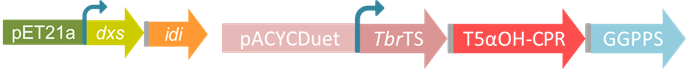 | p40T7-*dxs-idi*,  pACYC-ED5 | 11 | 2.9 | 1.3 |
| TaolED6 | 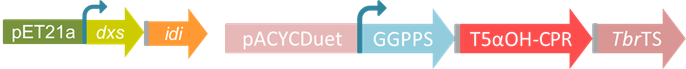 | p40T7-*dxs-idi*,  pACYC-ED6 | 1.4 | 0.40 | 0.18 |
| TaolV1 | 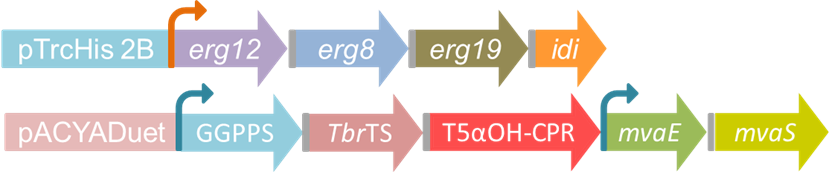 | TrcE,  AES4 | 14 **^b^** | 3.8 **^b^** | 1.3 **^b^** |
| TaolV2 | 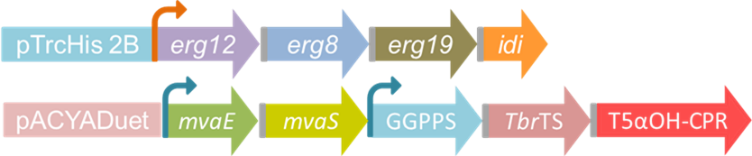 | TrcE,  AES5 | 3.5 | 0.62 | 0.57 |
| TaolV3 | 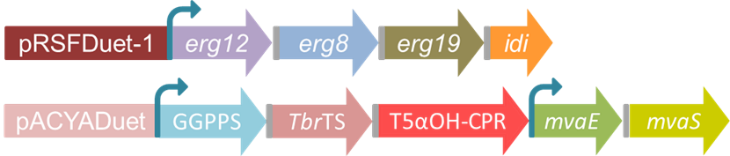 | T7E1,  AES4 | 5.9 | 1.7 | 0.67 |
| TaolV4 | 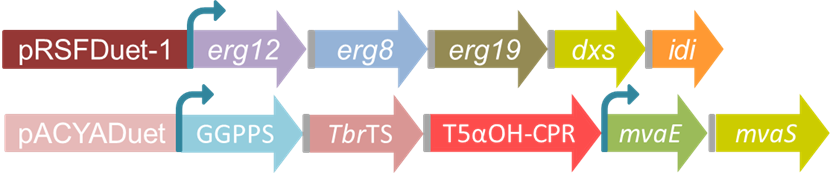 | T7E2,  AES4 | 9.1 | 2.2 | 1.1 |
| TaolV5 | 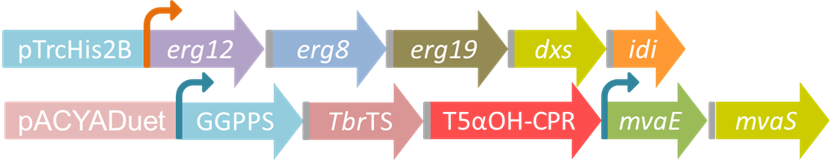 | TrcE1,  AES4 | 5.1 | 1.7 | 0.80 |

**^a^**: All experiments were performed for triplicate, with error bars showing the standard deviations; **^b^**: None of the data in the table has been optimized for culture conditions.

**Table S2** The primers used in this study.

| Name | Sequence (5’ to 3’) |
| --- | --- |
| TG-MF | CTTTAATAAGGAGATATACCATGAGCAGCAGCACCGGCAC |
| TG-MR | GTGGCAGCAGCCTAGGTTAATCACAACTGACGAAACGCAA |
| TG-ZF | GTGCCGGTGCTGCTGCTCATGGTATATCTCCTTATTAAAG |
| TG-ZR | TTGCGTTTCGTCAGTTGTGATTAACCTAGGCTGCTGCCAC |
| T5-MF | TTAATAAGGAGATATACCATGGCGCTGCTGCTGGCGGTTTTCTTTAGCATTGCGCTGAGCGCGATTGCTG |
| T5-MR | CTGGGTATCGGAACCGCCGCGACGGGATCCGGTGCTACCCGGACGCGGAAACAGCTTGATGCT |
| CPR-MF | AGCATCAAGCTGTTTCCGCGTCCGGGTAGCACCGGATCCCGTCGCGGCGGTTCCGATACCCAG |
| CPR-MR | GCCATATGTATATCTCCTTCTTTTACCAGATGTCACGCAGGT |
| TC-ZF | CAGCAATCGCGCTCAGCGCAATGCTAAAGAAAACCGCCAGCAGCAGCGCCATGGTATATCTCCTTATTAA |
| TC-ZR | ACCTGCGTGACATCTGGTAAAAGAAGGAGATATACATATGGC |
| Tba-MF | GTTTAACTTTAATAAGGAGATATACCATGGCAATGAGCAGCAGCAGCAC |
| Tba-MR | CCGAGCTCGAATTCGGATCCTTACACCTGAATCGGGTCGA |
| Tba-ZF | GTGCTGCTGCTCATTGCCATGGTATATCTCCTTATTAAAGTTAAAC |
| Tba-ZR | TCGACCCGATTCAGGTGTAAGGATCCGAATTCGAGCTCGG |
| Tw-MF | GTTTAACTTTAATAAGGAGATATACCATGGCAATGAGCAGCAGCAC |
| Tw-MR | CCGAGCTCGAATTCGGATCCTTAAACCTGAATCGGGTCGA |
| Tw-ZF | GTGCTGCTCATTGCCATGGTATATCTCCTTATTAAAGTTAAAC |
| Tw-ZR | TCGACCCGATTCAGGTTTAAGGATCCGAATTCGAGCTCGG |
| ATR-MF | TTTAACTTTAATAAGGAGATATACCATGGCGCTGCTGCTGGCGGTTTTCTTTAGCATTGCGCTGAGCGCG |
| ATR-MR | ATATGTATATCTCCTTCTTTTACCATACATCTCTAAGATAT |
| ATR-ZF | CGCGCTCAGCGCAATGCTAAAGAAAACCGCCAGCAGCAGCGCCATGGTATATCTCCTTATTAAAGTTAAA |
| ATR-ZR | ATATCTTAGAGATGTATGGTAAAAGAAGGAGATATACATAT |
| G1-MF | TCAAGCTGTTTCCGCGTCCGGGATCCGGACGTCGCGGCGGTTCCGATAC |
| Gn-MR | TGACAATCGGGGTGGTGCTGTCCGCCGGCA |
| G1-ZF | GTATCGGAACCGCCGCGACGTCCGGATCCCGGACGCGGAAACAGCTTG |
| Gn-ZR | TGCCGGCGGACAGCACCACCCCGATTGTCA |
| G2-MF | TCAAGCTGTTTCCGCGTCCGGGATCCGGAGGATCCGGACGTCGCGGCGGTTCCGATAC |
| G2-ZF | GTATCGGAACCGCCGCGACGTCCGGATCCTCCGGATCCCGGACGCGGAAACAGCTTGA |
| G3-MF | TCAAGCTGTTTCCGCGTCCGGGATCCGGAGGATCCGGAGGATCCGGACGTCGCGGCGGTTCCGATAC |
| G3-ZF | GTATCGGAACCGCCGCGACGTCCGGATCCTCCGGATCCTCCGGATCCCGGACGCGGAAACAGCTTGA |
| G4-MF | TCAAGCTGTTTCCGCGTCCGGGATCCGGAGGATCCGGAGGATCCGGAGGATCCGGACGTCGCGGCGGTTCCGATAC |
| G4-ZF | GTATCGGAACCGCCGCGACGTCCGGATCCTCCGGATCCTCCGGATCCTCCGGATCCCGGACGCGGAAACAGCTTGA |
| G5-MF | TCAAGCTGTTTCCGCGTCCGGGATCCGGAGGATCCGGAGGATCCGGAGGATCCGGAGGATCCGGACGTCGCGGCGGTTCCGATAC |
| G5-ZF | GTATCGGAACCGCCGCGACGTCCGGATCCTCCGGATCCTCCGGATCCTCCGGATCCTCCGGATCCCGGACGCGGAAACAGCTTGA |
| 1-G-MF | ACCTGCGTGACATCTGGTAATTTAATAAGGAGATATACCATGTTTGATTTCAATGAATA |
| 1-G-MR | GTGCCGGTGCTGCTGCTCATGGTATATCTCCTTATTAAATCACAACTGACGAAACGCAA |
| 1-TS-MF | TTGCGTTTCGTCAGTTGTGATTTAATAAGGAGATATACCATGAGCAGCAGCACCGGCAC |
| 1-TS-MR | AAGCTGCGCTAGTAGACGAGTCCATTTAAACCTGAATCGGATCGA |
| 1-ZF | TTACCAGATGTCACGCAGGT |
| 1-ZR | ATGGACTCGTCTACTAGCGCAGCTT |
| 2-G-MF | ACCTGCGTGACATCTGGTAATTTAATAAGGAGATATACCATGAGCAGCAGCACCGGCAC |
| 2-G-MR | TATTCATTGAAATCAAACATGGTATATCTCCTTATTAAATTAAACCTGAATCGGATCGA |
| 2-TS-MF | TCGATCCGATTCAGGTTTAATTTAATAAGGAGATATACCATGTTTGATTTCAATGAATA |
| 2-TS-MR | AAGCTGCGCTAGTAGACGAGTCCATTCACAACTGACGAAACGCAA |
| 2-ZF | TTACCAGATGTCACGCAGGT |
| 2-ZR | ATGGACTCGTCTACTAGCGCAGCTT |
| 3-G-MF | TCGATCCGATTCAGGTTTAATTTAATAAGGAGATATACCATGTTTGATTTCAATGAATA |
| 3-G-MR | AGCAGCGCCATGGTATATCTCCTTATTAAATCACAACTGACGAAACGCAATGTAATCGGC |
| 3-TS-MF | TAACAATTCCCCTGTAGAAATTTAATAAGGAGATATACCATGAGCAGCAGCACCGGCACCAGCAAGGTG |
| 3-TS-MR | TATTCATTGAAATCAAACATGGTATATCTCCTTATTAAATTAAACCTGAATCGGATCGA |
| 3-ZF | TACAGGGGAATTGTTATCCG |
| 3-ZR | TTTAATAAGGAGATATACCATGGCGCTGCTGCT |
| 4-T5-MF | TCGATCCGATTCAGGTTTAATTTAATAAGGAGATATACCATGGCGCTGCTGCTGGCGGT |
| 4-T5-MR | TATTCATTGAAATCAAACATGGTATATCTCCTTATTAAATTACCAGATGTCACGCAGGT |
| 4-G-MF | ACCTGCGTGACATCTGGTAATTTAATAAGGAGATATACCATGTTTGATTTCAATGAATA |
| 4-G-MR | AGCCTAGGTTAATTAAGCTGTCACAACTGACGAAACGCAA |
| 4-ZF | TTAAACCTGAATCGGATCGA |
| 4-ZR | CAGCTTAATTAACCTAGGCT |
| 5-T5-MF | TCGATCCGATTCAGGTTTAATTTAATAAGGAGATATACCATGGCGCTGCTGCTGGCGGT |
| 5-T5-MR | TATTCATTGAAATCAAACATGGTATATCTCCTTATTAAATTACCAGATGTCACGCAGGT |
| 5-G-MF | ACCTGCGTGACATCTGGTAATTTAATAAGGAGATATACCATGTTTGATTTCAATGAATA |
| 5-G-MR | AGCCTAGGTTAATTAAGCTGTCACAACTGACGAAACGCAA |
| 5-ZF | TTAAACCTGAATCGGATCGA |
| 5-ZR | CAGCTTAATTAACCTAGGCT |
| 6-T5-MF | TTGCGTTTCGTCAGTTGTGATTTAATAAGGAGATATACCATGGCGCTGCTGCTGGCGGT |
| 6-T5-MR | GTGCCGGTGCTGCTGCTCATGGTATATCTCCTTATTAAATTACCAGATGTCACGCAGGT |
| 6-G-MF | CTTTAATAAGGAGATATACCATGTTTGATTTCAATGAATA |
| 6-G-MR | ACCGCCAGCAGCAGCGCCATGGTATATCTCCTTATTAAATCACAACTGACGAAACGCAA |
| 6-TS-MF | ACCTGCGTGACATCTGGTAATTTAATAAGGAGATATACCATGAGCAGCAGCACCGGCAC |
| 6-TS-MR | CGATTACTTTCTGTTCGACTTTAAACCTGAATCGGATCGA |
| 6-ZF | GGTATATCTCCTTATTAAAGTTAAACAAAATTATTT |
| 6-ZR | AGTCGAACAGAAAGTAATCGTATTGTACACGGCCGCA |
| E12-MF | GTATCGATTAAATAAGGAGGAATAAACCATGAGTCTACCATTTTTAACATCAGCTCCC |
| E12-MR | TTAGCTGGTCACGGCAG |
| E8-MF | CTGCCGTGGACCAGCTAAGTATAAGAGGAGGTAAAAAAACATGTCAGAACTAAGGGCATTTAGTGC |
| E8-MR | CGCTCGCCTTGTAAACGATCTTATTTGTCCAGATACGTTTCCGGGTC |
| MVA1-ZF | GGTTTATTCCTCCTTATTTAATCGATACATTAATATATACC |
| MVA1-ZR | GATCGTTTACAAGGCGAGCG |
| E19-MF | GACCCGGAAACGTATCTGGACAAATAAGTATAAGAGGAGGTAAAAAAACATGGCAATGACAGTTTATACGGC |
| E19-MR | TTACTCTTTCGGCAGACCGG |
| I-MF | CCGGTCTGCCGAAAGAGTAAGTATAAGAGGAGGTAAAAAAACATGACAGCTGATAATAACTCAATGCCC |
| I-MR | CCGTTTAAACTCAATGATGATGATGATGATGTTACAGCATACGATGAATCTGACGATCG |
| MVA2-ZF | CATCATCATCATCATCATTGAGTTTAAACGG |
| MVA2-ZR | TTATTTGTCCAGATACGTTTCCGGGTC |
| E-MF | CTTTAATAAGGAGATATACCATGAAAACAGTTGTAATAATTGATGCTCTA |
| E-MR | TTACTGTTTGCGCAGGTCGTTC |
| S-MF | GAACGACCTGCGCAAACAGTAAGTATAAGAGGAGGTAAAAAAACATGACAATTGGAATAGATAAAATATCATTTTTCG |
| S-MR | GCGCCGAGCTCGAATTCGGATCCTTAATTGCGGTAGCTACGAACGG |
| AES-ZF | GGTATATCTCCTTATTAAAGTTAAACAAAATTATTTCTACAGGGG |
| AES-ZR | GGATCCGAATTCGAGCTCGGCGCGCCTGCA |
| 4-MF | GATATACATATGGCAGATCTATGAAAACAGTTGTAATAAT |
| 4-MR | CCAGACTCGAGGGTACCGACTTAATTGCGGTAGCTACGAA |
| AES4-ZF | ATTATTACAACTGTTTTCATAGATCTGCCATATGTATATC |
| AES4-ZR | TTCGTAGCTACCGCAATTAAGTCGGTACCCTCGAGTCTGG |
| 5-MF | TAGTTAAGTATAAGAAGGAGATATACATATGTTTGATTTCAATGAATA |
| 5-MR | CGGTTTCTTTACCAGACTCGAGTTACCAGATGTCACGCAGGT |
| AES5-ZF | TATTCATTGAAATCAAACATATGTATATCTCCTTCTTATACTTAACTA |
| AES5-ZR | ACCTGCGTGACATCTGGTAACTCGAGTCTGGTAAAGAAACCG |
| ET7-MF | CTTTAATAAGGAGATATACCATGAGTCTACCATTTTTAAC |
| ET7-MR | TTATGCGGCCGCAAGCTTGTTTACAGCATACGATGAATCT |
| ET7-ZF | GTTAAAAATGGTAGACTCATGGTATATCTCCTTATTAAAG |
| ET7-ZR | AGATTCATCGTATGCTGTAAACAAGCTTGCGGCCGCATAA |
| D4-MF | CCGGTCTGCCGAAAGAGTAAGTATAAGAGGAGGTAAAAAAACATGAGTTTTGATATTGCCAA |
| D4-MR | GAGTTATTATCAGCTGTCATATGTATATCTCCTTCTTATGCCAGCCAGGCCTTGAT |
| D4-ZF | TTGGCAATATCAAAACTCATGTTTTTTTACCTCCTCTTATACTTACTCTTTCGGCAGACCGG |
| D4-ZR | ATCAAGGCCTGGCTGGCATAAGAAGGAGATATACATATGACAGCTGATAATAACTC |
| D5-MF | GGTCTGCCGAAAGAGTAAGTATAAGAGGAGGTAAAAAAACATGAGTTTTGATATTGCCAAATACCCGACC |
| D5-MR | TTGAGTTATTATCAGCTGTCATGTTTTTTTACCTCCTCTTATACCTTATGCCAGCCAGGCCTTGATTTTG |
| D5-ZF | CTCCTCTTATACTTACTCTTTCGGCAGACC |
| D5-ZR | ATGACAGCTGATAATAACTCAATGCCCCACGGTGC |
